# Cross-National Patterns of Intergenerational Continuities in Childbearing in Developed Countries

**DOI:** 10.1080/19485565.2013.833779

**Published:** 2013-11-12

**Authors:** Michael Murphy

**Affiliations:** ^a^ Department of Social Policy, London School of Economics, London, United Kingdom

## Abstract

Earlier work has shown that the association between the fertility of parents and the fertility of children has become stronger over time in some societies. This article updates and broadens the geographic coverage to assess the magnitude of intergenerational continuities in childbearing in developed and middle-income societies using data for 46 populations from 28 developed countries drawn from a number of recent large-scale survey programs. Robust positive intergenerational fertility correlations are found across these countries into the most recent period, and although there is no indication that the strength of the relationship is declining, the increasing trend does not appear to be continuing.

## Introduction

Fertility patterns of parents and their children tend to be positively correlated. Results comparing the completed fertility of successive generations for both historical and more recent populations in a number of now-developed countries up to the later part of the twentieth century are presented in Murphy (1999). This article updates and considerably broadens the geographic coverage to assess the magnitude of intergenerational continuities in childbearing in a number of developed and middle-income societies and, in particular, to establish whether earlier trends have continued into the most recent period. It also considers the relationship with macro-level factors and how much the relationship changes when socioeconomic controls are included.

The association between parents' fertility and their children's fertility has been a subject of scientific interest since the late nineteenth century. Pearson, Lee, and Bramley-Moore (1899) investigated the extent of fertility correlations among branches of the British upper classes, one of the few sources of such data at that time. They found the highest correlation coefficient of 0.21 for the fertility of 1,000 mother-daughter pairs from the peerage and baronetage when both had marriages that had lasted for at least 15 years. The lowest reported correlation coefficient for women was 0.04 for 4,418 pairs of mothers and daughters drawn from a mixture of upper-class groups, with the only restriction being that the daughter should have been married for 15 years or had a spouse who died before 15 years of marriage.

### Early Populations

Pearson, Lee, and Bramley-Moore (1899:279) assumed that the mechanism of transmission was biological rather than social and that their study populations undertook no deliberate attempt to limit fertility, although they did suggest that fertility control would tend to depress correlations. Studies of less specialized pretransitional historical populations are rare. Langford and Wilson (1985) analyzed 10,931 English ever-married mother-daughter pairs from the mid-sixteenth to mid-nineteenth centuries. They computed a small intergenerational fertility correlation coefficient of 0.02 and concluded that there was no association between the fertility of mothers and the fertility of daughters. Bocquet-Appel and Jakobi (1993) estimated a similar correlation of 0.015 for fertility of 257 pairs of once-married women in successive generations in a French village in the early part of the nineteenth century. They also rejected the existence of intergenerational transmission of fertility in this pretransitional period. Neel and Schull (1972:345) analyzed a rather later population, but one that did not use birth control—Amish couples who married in the period 1820–1879 in Ohio and Indiana—and they computed slightly higher intergenerational correlations between their fertility and the sibship sizes of wives and husbands of 0.09 and 0.07, respectively.

Recently, biological interest in fertility transmission has revived, in part because of its importance for transmission of rare genetic diseases (Tremblay 1997; Austerlitz and Heyer 1998; Gagnon and Heyer 2001; Helgason et al. 2003). Such studies typically analyze the intergenerational fertility patterns of descendants of an initial population over extended periods of time. Based on analyses of two Québec populations, Gagnon and Heyer (2001) also concluded that family size does not have a tendency to run in families. However, much higher correlation coefficients of 0.31 (*p* < 10^−6^) for completed fertility between couples and their sons and of 0.23 (*p* < .001) between couples and their daughters were found for a set of 161 genealogies in the Hutterite archetypal natural fertility population (Pluzhnikov et al. 2007). These results cover an extended time period, and so they were adjusted to control for time trend. They show a high correlation in a well-documented and socioeconomically and behaviorally homogeneous population.

Gagnon and Heyer (2001) found a much stronger association between the effective family sizes of “settled populations” in Québec—that is, populations in which both successive generations remained in the study area and survived to be involved in procreation—a result that they attributed mainly to sociodemographic factors rather than biological transmission (Heyer, Sibert, and Austerlitz 2005). In contemporary developed societies, the difference between numbers of total and living children is small, so we are unable to address this issue, although for developing countries, where mortality levels are higher, the strength of the intergenerational associations is similar (Murphy 2012).

Studies concerned with biological reproductive performance or disease transmission are primarily interested in those individuals who are engaged in such activity, and many studies including those mentioned earlier are confined to married (sometimes further restricted to individuals in long-term, intact marriages) or parous individuals. The results are usually not representative of the overall population concerned, since, for example, they exclude immigrants who arrive during the analysis period. While findings from these pretransitional populations need to be interpreted carefully because of the selected nature of samples used, such populations tend to show positive but modest and statistically nonsignificant intergenerational fertility correlations that are usually only slightly larger than the early values of Langford and Wilson (1985) and Bocquet-Appel and Jakobi (1993).

Anderton et al. (1987) used data from the Utah Population Database to show a positive association between the fertility of women born in the period 1830 to 1870 and the fertility of their daughters, but the researchers did not present correlation coefficients. However, Jennings, Sullivan, and Hacker (2012) used the same data source and showed that the correlation coefficients for the fertility of women born between 1840 and 1899 and the fertility of their mothers and mothers-in-law (confined to those who were still married at age 45 in nonpolygamous unions) were 0.085 and 0.055, respectively. Studies of later populations often show higher values; Madrigal, Relethford, and Crawford (2003) found a correlation of 0.17 between mothers' and daughters' fertility in Ireland around the start of the twentieth century. However, as with many studies, this work was based on the comparison of successive generations of married women, so selection effects could be important. In this population, a substantial fraction of babies did not survive to adulthood, very high proportions of women never married, and emigration was widespread. If children from large families were less likely to have survived to adulthood, to be married, or to remain in Ireland, overall intergenerational correlations would be expected to be lower. Reher, Ortega, and Sanz-Gimeno's (2008) study of the Spanish town of Aranjuez in the period 1871–1970 was able to look at time trends. The correlation between fertility of mothers and fertility of daughters was 0.15 in the period 1871–1970, 0.19 in the period 1891–1910, and 0.14 in the period 1923–1945. These differences are not statistically significant, suggesting the persistence of similar levels of intergenerational continuities. Bresard (1950) used information on the number of children of the father and both grandfathers to examine the association between respondents' and their fathers' and mothers' number of siblings for a representative sample of 3,000 French men aged 18 to 50 in 1948 in a study that covers a similar childbearing period as that used by Reher, Ortega, and Sanz-Gimeno (2008). For the occupational group of farmers and peasants, who accounted for 34 percent of the sample, the correlation coefficient for the respondent's number of siblings and the maternal grandfather's number of children was 0.27; the correlation coefficient dropped to 0.24 for paternal grandfathers. These correlations are higher than those reported in earlier studies.

For a slightly later period of childbearing of the second generation, around the 1920s and 1930s, studies in Britain by Berent (1953) and in the United States by Kantner and Potter (1954) reported intergenerational correlations between mothers and daughters of 0.19 and 0.11, respectively. Both studies sampled surveys, although neither was completely random; Berent's (1953) results were based on 1,377 couples who were in their first marriage, with that marriage being of at least 15 years duration. He found no statistically significant differences between social classes and birth controllers/noncontrollers, suggesting similarity between these subgroups.

### More Recent Populations

Kantner and Potter (1954:295) argued that any intergenerational fertility transmission in modern populations is largely determined by the older generation forming notions or instilling preferences about family formation in the younger generation, but they found that such effects were, in any case, trivial. They also argued that with less variation in fertility levels, such effects would be likely to die out. However, Pullum and Wolf (1991) presented data on correlations between the number of living siblings and the number of children for several countries in a kin-modeling study. In the 1985 Canadian General Social Survey of five-year age groups ranging from 55–59 to 85 and over, the largest correlation coefficient of 0.32 was for women aged 60 to 64, with values declining at older ages (although since results were based on living siblings, the death of siblings would increasingly affect values).

Murphy (1999) used data from the 1976 British Family Formation Survey to show the relationship between the fertility of partnered women and the number of their own and their partner's siblings, and for all women, regardless of their partnership status with their siblings. The influences of husbands' and wives' family sizes were essentially equal, and there is no evidence of change in the strength of the relationship over time. However, his analysis of the 1987–1988 U.S. National Survey of Families and Households (NSFH) shows that the correlation coefficient for the respondent's number of children and his or her number of co-resident siblings during childhood increased for younger cohorts. For these data sources, the correlation coefficients are mostly in the range of 0.15 to 0.20.

The overall conclusion is that the relationship between the fertility of successive generations in developed countries tended to become stronger during the demographic transition until the latest period analyzed in the 1970s when the younger generation was bearing children.

### Disciplinary Perspectives

Intergenerational continuities in age at childbearing have been studied in a number of disciplines, which generally find a positive correlation between parents' and their children's age at first birth (Barber 2000; Barber 2001; Stanfors and Scott 2013 Steenhof and Liefbroer 2008; van Bavel and Kok 2009). The long-standing interests of demographers and historians in this subject have continued, with special editions of *Human Nature* and a recent volume of *History of the Family* dedicated to the topic of intergenerational transmission (Bittles, Murphy, and Reher 2008; Murphy 2013). The topic of intergenerational fertility continuities among immigrants is of particular policy interest (Nosaka and Chasiotis 2010; Parrado and Morgan 2008; Scott and Stanfors 2011) In social policy and public health, a number of studies have focused on the intergenerational transmission of teenage motherhood (Furstenberg, Levine, and Brooks-Gunn 1990; Horwitz et al. 1991; Kahn and Anderson 1992; Manlove 1997). These studies show that children born to young mothers are at higher risk of having their first child at a young age, and that siblings' behavior also has an effect. This topic has also become of interest to economists (e.g., Booth and Kee 2009), although the underlying model in that discipline is the long-standing family-specific “cultural transmission” model that dominated U.S. sociological thinking in the 1960s and 1970s.

The relationship between childbearing and the fertility of siblings and other close kin has recently become of wider interest, although it had previously been considered by Imaizumi, Nei, and Furusho (1970) and Bocquet-Appel and Jakobi (1993). If parents' and children's fertility is correlated, then siblings' fertility must also be. However, demographic studies of Scandinavian registers (which are one of the rare large-scale accurate sources of such data) for Denmark by Murphy and Knudsen (2009) and for Norway by Lyngstad and Prskawetz (2010) show that there is an independent effect of siblings over and above the indirect parental effect. Murphy and Wang (2001) used the NSFH data to show that grandparental fertility has an independent additional impact on individuals' fertility, indicating that such effects span multiple generations and potentially have more impact on long-term demographic trends. The independent contribution of grandparental fertility to that of their grandchildren was confirmed by Kolk (2013) using Swedish register data; Kolk also found that intergenerational correlations between parents and children are positive and stronger with daughters than with sons. However, the younger generation in this study was confined to those born in the period 1970–1982, who have not completed childbearing, so the results are not directly comparable with those of analyses of completed fertility.

There has been a resurgence of interest in biological, especially genetic, studies, and other disciplines have also become interested in intergenerational transmission in the context of wider kin influences. The importance of wider kin networks' pro-natalist social influence and instrumental help has been stressed (Balbo and Mills 2011; Bernardi and White 2010; Kaptijn et al. 2010; Lyngstad and Prskawetz 2010; Matthews and Sear 2013; Newson and Richerson 2009). These include evolutionary interests in the impact of “cooperative breeding” and “helpers at the nest” (Kramer 2010).

Even if variables related to socialization factors, including stability of lifestyle and childhood satisfaction, are related to subsequent fertility, this would not mean that genetic factors are ruled out, since these pathways may be genetically influenced. More specialized designs that permit the relative contribution of genes, environment, and their interaction to be determined (such as twin or adoption studies) are required. Studies by Kohler, Rogers, and Christensen (1999) based on Danish twins born in the period 1870–1964 and by Bras, Van Bavel and Mandemakers (2013) using the GENLIAS database for sibling pairs born in Zeeland in the period 1812–1866 suggest a substantial genetic component to fertility. Guo and Tong (2006) used a different approach, DNA analysis, to identify polymorphisms associated with early initiation of sexual intercourse.

### Beyond Overall Correlations

A key issue is the extent to which simple intergenerational correlations are attenuated or even eliminated by controlling for socioeconomic factors. Pearson, Lee, and Bramley-Moore (1899:277) distinguished between intergenerational correlations that can arise because of the transmission of fertility between parents and children and “spurious” ones that result from the mixing of heterogeneous populations. This point became particularly pertinent when Williams and Williams (1974) showed that even simple disaggregation by time period of Pearson, Lee, and Bramley-Moore's father-son pairs, results led to very different findings using essentially the same data. Their overall value of 0.06 for fathers and sons was almost identical to Pearson, Lee, and Bramley-Moore's value of 0.07, but they also computed correlation coefficients for three different generations. These fell from 0.17 to 0.04 to 0.02 over three grandparental generations born in the years 1740 to 1800. Thus, over time, they found that the relationship between male sibship size and fertility became weaker. Williams and Williams argued that the social environment is the overwhelming determinant of fertility rather than “biology.” They attributed the reduced correlations to the fact that the environment is becoming more changeable over time (although a range of studies including some discussed previously find that the strength of the relationship increased over the demographic transition).

Some studies have attempted to control for heterogeneity—for example, by dividing populations into more homogenous units or by restricting analysis to particular subpopulations. However, the availability of micro-level data means that multivariate analysis can be used to control for population heterogeneity more efficiently. Murphy and Wang (2001) investigated how far controlling for socioeconomic factors such as education level altered the conclusions using multilevel models for populations in a small number of countries. They used the 1986 International Social Survey Programme (ISSP) data on social networks; data for Italy, Norway, and Poland from the United Nations Economic Commission for Europe (UNECE) Fertility and Family Survey (FFS) program; and the U.S. NSFH. They concluded that the intergenerational relationship cannot be explained by differential fertility across socioeconomic groups. Murphy and Knudsen (2002) used register data from Denmark to show that intergenerational continuities are robust to the inclusion of both socioeconomic variables and birth order as possibly confounding factors. However, full linkage of the younger generation to their parents was only available up to about age 26, so comparisons of completed fertility of successive generations are not possible.

Analyses over the past 100 years on the association between fertility of parents and fertility of children have highlighted a number of issues that will be considered here. One is whether the increase in intergenerational fertility correlations has continued for the most recent cohorts when fertility has been relatively stable. There are two reasons why an increase is particularly likely to be observed when fertility changes rapidly. First, there could be greater heterogeneity between groups in the adoption of fertility control, which would be likely to lead to intergenerational correlations if fertility differed, for example, between urban and rural areas or socioeconomic groups. Second, at such periods, individuals may have greater choice regarding their family-building decisions, whereas earlier and later periods are characterized by greater homogeneity (Bras, Van Bavel, and Mandemakers 2013; Jennings, Sullivan, and Hacker 2012; Kohler, Rogers, and Christensen 1999; Reher, Ortega, and Sanz-Gimeno 2008). On the other hand, the greater levels of individual autonomy and control in modern developed societies might enable women to match their own outcome more closely to that of their family of orientation. A related question is what factors are associated with high or low intergenerational fertility continuities in different parts of the developed world. These issues form the focus of the remainder of the article. I start by describing the data sources used.

## Data and Methods

### Data

The data sources used are as follows:

#### Fertility and Family Surveys (FFS)

During the 1990s, 24 countries participated in the UNECE FFS project using a standardized questionnaire. Countries complied with this standard questionnaire to different degrees in their national instruments. There was a minimal core questionnaire and optional modules, so information on siblings is available for only 11 countries: Austria, Canada, Finland, Hungary, Italy, Latvia, Lithuania, Norway, Poland, Slovenia, and Switzerland. The question used was: “Including yourself, how many children has your mother had in all, who were born alive?” Further information is available at http://www.unece.org/fileadmin/DAM/pau/_docs/ffs/FFS_2000_Prog_SurveyDesign.pdf.

#### International Social Survey Programme (ISSP)

The 2001 round of the ISSP collected information on social relations and social networks, replicating the 1986 round that included a much smaller number of countries. Topics included number of adult brothers and sisters, as well as frequency of contact with parents, brothers and sisters, and own children. It also collected a range of other information, including respondents' number of children younger than 18 years, number of children 18 or older, age, education, religious denomination, and church service attendance. An example of question wording was as follows: “How many adult brothers and/or sisters—we mean brothers or sisters who are age 18 and older—do you have? (We mean brothers and sisters who are still alive. Please include step-brothers and -sisters, half-brothers and -sisters and adopted brothers and sisters).”

However, a number of countries did not include the question on number of younger children. Some countries did not collect information about number of siblings, and in some cases, the proportion of missing cases was substantial, so I excluded countries with more than 15 percent of cases missing. Twenty ISSP surveys that included questions on siblings and all children are analyzed here: those for Austria, Canada, Chile, Cyprus, Czech Republic, Denmark, France, Germany (West and East separately, as the countries were separate when childbearing was largely taking place), Hungary, Italy, Japan, Latvia, Northern Ireland (the question on younger children was not included in the rest of the United Kingdom), Poland, the Russian Federation, Slovenia, Spain, Switzerlandm and the United States. For more information, see http://www.issp.org/.

#### Generations and Gender Programme (GGP)

This set of panel surveys of representative samples of people aged 18 to 79 is conducted by the UNECE. The surveys collect information on a range of socioeconomic topics, including social networks and education. Information was collected on total and living siblings in a subset of countries and was made available in early 2012 for 13 countries: Austria, Belgium, Bulgaria, Estonia, France, Georgia, Germany, Italy, Lithuania, the Netherlands, Norway, Romania, and the Russian Federation; some results are also available for the special German Turkish sample. Questions include both how many brothers and sisters the respondent ever had and how many are currently alive, including full, half, adopted, and foster siblings. For further details, see http://www.unece.org/pau/ggp/welcome.html.

#### Understanding Society (UNSOC)

Understanding Society is a panel study of the socioeconomic circumstances and attitudes of 100,000 individuals in 40,000 British households. The survey collects information on the total number of siblings and children living both in the household and elsewhere. The sample size and detail of information available is more substantial than that for the previously mentioned surveys conducted by international programs, which in any case do not include Great Britain. For more information, see http://www.understandingsociety.org.uk/.

The main indicator of socioeconomic status available in all surveys is the education level of the respondent from the younger generation (the education level of the older generation is not generally available), with the exception of Northern Ireland (in the ISSP) and France (in the GGP). The three international data sources code education as consistently as possible within each set, but they are not consistently coded across sets, so we use them principally as control variables to assess how far their inclusion alters the magnitude of sibling coefficients, rather than the magnitude of the specific education level per se. The FFS and GGP data include a relatively homogenous set of countries, so the highest recorded level is based on the UNESCO International Standard Classification of Education (ISCED), although the classification was updated between the two surveys. UNSOC uses a classification based on the highest examination level achieved that does not map directly into ISCED but also produces an ordinal scale. However, since the ISSP includes wider geographical and developmental ranges, the education variable is based on tertiles of years of education within each country, age, and sex group, that is, it indicates the level relative to similar individuals.

Although the effect of religious denomination on fertility is weakening, religious participation remains a strong predictor (McQuillan 2004). The ISSP collects information not only on religious identification, but also on commitment, measured in terms of frequency of attendance at services. This may be regarded as an indicator of more traditional attitudes, and therefore those who are more committed may be more likely to follow parents' patterns.

The FFS is largely confined to respondents younger than age 50 (with a small number of respondents over this age), and the GGP to those younger than age 80. The other sources have no upper age limit, although relatively few respondents are older than age 75 (15 percent in the ISSP, 9 percent in the GGP, and 18 percent in the UNSOC among those aged 40 and over). Basic descriptive data for the surveys included are given in Appendix A.

I also present national-level comparisons, since it is possible that international patterns may be very different from intranational ones, as is the case for mortality and income (at least in highly developed societies; see, e.g., Preston, 2007). For macro-level data, I used four main indicators:

• Life expectancy at birth
• Real gross national income per head (GNI)
• Average number of siblings among respondents aged 40 and over
• Average number of children among respondents aged 40 and over

The first two indicators are key components of the UN Human Development Index (HDI) and are the latest values in the 2011 HDI Report (see http://hdr.undp.org/en/statistics/hdi/), rather than those from around the time of childbearing, but they indicate the general relative positions of the countries. The last two values are indicators of fertility of the older and younger generations, computed as national average values from the surveys analyzed here and shown in Appendix A.

### Methods

Individual-level data from the sources described previously considerably extend the sample sizes, availability of covariates, and range of countries for which data are available. These micro-level data allow multivariate analysis of how far intergenerational continuities in childbearing vary not only across countries but also between socioeconomic groups, such as by education level, to assess how far such variables may attenuate the strength of the relationship. However, until recently, the principal indicator available for comparisons was Pearson product moment correlation coefficients for the fertility of successive generations (usually based on respondents' sibship size and number of children), which are needed for comparisons with earlier studies, so I also included analyses based on these measures.

In order to assess the effect of including additional covariates, to allow for overdispersion, I fit two sets of quasipoisson generalized linear models to respondents aged 40 and over from these four data sets. The first set of four increasingly detailed models is as follows:

1. ln(*Children_ij_*) = μ + α *Country_j_* + β *Age_ij_* + γ *Sex_ij_* + ϵ*_ij_*
2. ln(*Children_ij_*) = μ + α *Country_j_* + β *Age_ij_* + γ *Sex_ij_* + δ *Sibs_ij_* + ϵ*_ij_*
3. ln(*Children_ij_*) = μ + α *Country_j_* + β *Age_ij_* + γ *Sex_ij_* + δ *Sibs_ij_* + θ *Education_ij_* + ϵ*_ij_*
4. ln(*Children_ij_*) = μ + α *Country_j_* + β *Age_ij_* + γ *Sex_ij_* + δ *Sibs_ij_* + θ *Education_ij_* + λ Sibs_ij_* *Sex_ij_* + ϵ*_ij_*

Where for respondent *i* living in country *j*, μ is the intercept term, *Children_ij_* is the number of respondent's children, *Age_ij_* is age (centered at age 40 and measured in decades) of respondent, *Sex_ij_* is sex of respondent, *Education_ij_* is highest education level of respondent, *Sibs_ij_* is number of siblings of respondent, *Country_j_* is the indicator for country *j*, and ϵ*_ij_* is an error term. Note that ISSP models also include the religious commitment variable in addition to education and that UNSOC does not include country as a covariate.

The second set of analyses fits two quasipoisson models without and with the education variables (and religious commitment for ISSP) to each separate population, with notation as previously outlined, apart from the superfluous country index:

1. ln(*Children_i_*) = μ + β *Age_i_* + γ *Sex_i_* +δ *Sibs_i_* + ϵ*_i_*
2. ln(*Children_i_*) = μ + β *Age_i_* + γ *Sex_i_* +δ *Sibs_i_* +θ *Education_i_* + ϵ*_i_*

## Results

The datasets used collected information on respondents' total number of children and number of siblings, although the precise form of the questions varied slightly. Results for overall correlation coefficients of the number of children of respondents aged 40 and over (who are assumed to have complete or almost complete fertility) and their number of siblings are shown in Figure 1, arranged by the three main datasets used (UNSOC data are also shown in Figure 1d). These are based on all cases for which information on both variables is available (sample sizes are given in Appendix A). Virtually all the correlations are positive, with the majority being in the range of 0.1 to 0.2 (the unweighted median across all countries shown in Figure 1d is 0.14), similar to the values reported for other recent studies. However, these patterns are now established to hold for a much wider set of countries than those from Western Europe and North America that are frequently analyzed.

**Figure:**
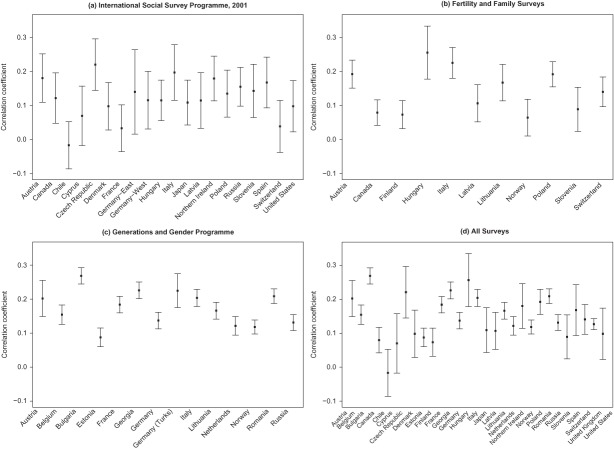
**Figure 1.** Correlation coefficients for number of respondents' siblings and children; respondents aged 40 and over.

Only one value per country is shown in Figure 1d, with the choice of survey for countries with multiple surveys in the following order: UNSOC (United Kingdom only), GGP, FFS, and ISSP, thus prioritizing the largest and most recent surveys. One country, Austria, is included in all three survey sets, although the cohorts covered are not identical. The values are very similar: 0.18 (± 0.036) for ISSP, 0.19 (± 0.021) for FFS, and 0.20 (± 0.027) for GGP. These values are not statistically significantly different from each other, suggesting that these data can be combined to provide a broader overview.

Only one value is negative, Chile (in ISSP), and that value is close to zero. ISSP sample sizes are smaller for each country than those used by the other surveys, and its results are based on living rather than ever-born siblings, but the number and geographical coverage of countries is wider than that of the other sets, including a number of middle-income countries that have not been analyzed in other studies so far. All the coefficients for the FFS, GGP, and UNSOC datasets are statistically significantly greater than zero at the 5 percent level, as are the great majority of ISSP values. The ISSP collects information on living siblings only, whereas the other surveys include all siblings. However, the GGP includes information on both living and total siblings, permitting comparison of the alternative indicators. The correlation between number of children and living siblings is 0.15 but reaches 0.18 for ever-born siblings, about one-fifth higher. The ISSP figures are therefore likely to produce slightly lower values than the other sources.

These results confirm that intergenerational fertility continuities remain positive for the most up-to-date data available for these mainly developed countries. However, there do not appear to be any obvious national-level factors that distinguish countries with particularly high or low values. Figure 2 shows a weakly negative relationship with two national-level indicators of human development, current life expectancy and real GNI per head (a nonparametric locally weighted scatterplot smoothing trend [Cleveland 1979] is also shown). The relationship between fertility of the two generations is also weakly negative; countries with relatively small average values in either generation tend to have higher correlations, suggesting that the cross-sectional relationship is not associated with high levels of fertility (Figures 2c and 2d). Therefore, although correlations tend to increase with “modernization” across time, the cross-sectional gradient is in the opposite direction. While global geographical coverage among these high- and middle-income countries is not complete, there is some evidence of regional clustering. The largest values are mainly concentrated in countries of southern and eastern Europe, including Austria, Italy, Spain, Hungary, Bulgaria, and Romania (see Table 1), members of the “strong family” system group (Reher 1998) that shows less modern family forms (Murphy 2008). Nordic and North American countries with “weaker family” systems have generally low values. Strong family ties may be a response to or a cause of relatively weak wider societal influences. These results are consistent with findings that intergeneration transmission tends to be stronger among groups for whom family influences and social controls are weak (Van Bavel and Kok 2009). The main conclusion is that positive intergenerational correlation is a general phenomenon across these countries, with little indication of variation by socioeconomic or demographic regime.

**Figure:**
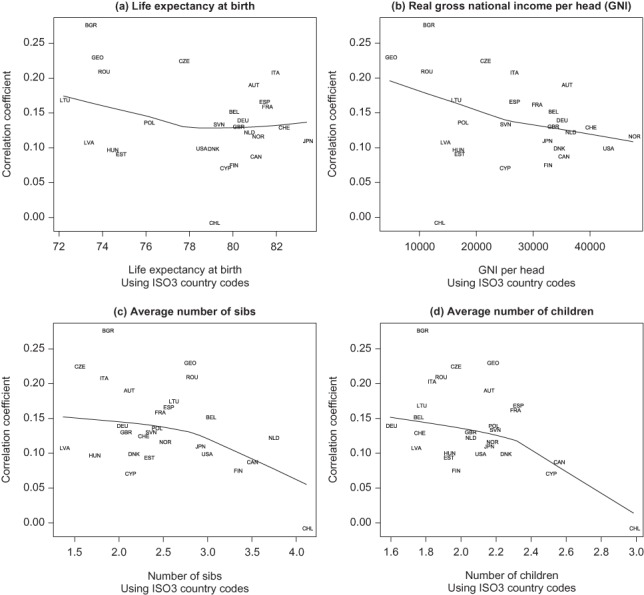
**Figure 2.** Relationship of national-level intergenerational correlation coefficients of respondents aged 40 and over with macro-level indicators.

**Table 1:** Average national correlation coefficients (unweighted) by United Nations global regional level

Region	Correlation	*N*
Eastern Europe	0.19	5
Northern Europe	0.11	7
Southern Europe	0.17	3
Western Europe	0.15	6
Northern America	0.09	2
*Notes:* Based on the standard UN regional classification (esa.un.org/wpp/Excel-Data/country-classification.pdf). There were also values for Eastern Asia (0.11; *N* = 1), Western Asia (0.15; *N* = 2), and South America (–0.01; *N* = 1).*Source:* Based on Fertility and Family Surveys (FFS), International Social Survey Programme (ISSP), Generations and Gender Programme (GGP), and Understanding Society Survey (UNSOC).		

The second issue that we consider is the role of individual-level covariates in relation to the effect of parental characteristics, including fertility. Fertility is influenced by a number of factors, such as religion and education, in addition to the size of family of orientation. There is a clear monotonic relationship between completed fertility and simple overall measures of both sibship size and highest education level (Table 2).

**Table 2:** Average number of children by (a) number of siblings and (b) education level

(a)	Number of siblings					
Survey	0	1	2	3	4	5 or more
ISSP (*N* = 14,242)						
Mean	1.88	1.96	2.16	2.25	2.28	2.64
*N*	2,310	3,576	3,101	1,993	1,231	2,031
FFS (*N* = 18,991)						
Mean	1.69	1.79	1.89	2.02	2.15	2.20
*N*	1,702	4,271	4,230	3,051	1,957	3,780
GGP (*N* = 78,118)						
Mean	1.50	1.67	1.82	1.98	2.08	2.23
*N*	8,300	19,728	17,378	11,812	7,562	13,338
UNSOC (*N* = 13,828)						
Mean	1.97	1.87	2.01	2.11	2.33	2.45
*N*	2,814	3,877	2,902	1,753	1,007	1,475

(b)	Educational classification					
Survey	Tertiles	Bottom	Middle	Top		
ISSP (*N* = 12,617)	Mean	2.34	2.08	1.94		
	*N*	4,594	3,885	4,138		
			Lower	Upper	Postsecondary	
	ISCED 1976	Primary	secondary	secondary	nontertiary	Tertiary
FFS (*N* = 18,011)	Mean	2.28	2.06	1.89	1.92	1.78
	*N*	2,602	3,519	7,862	1,847	2,181
			Lower	Upper	Postsecondary	
	ISCED 1995	Primary	secondary	secondary	nontertiary	Tertiary
GGP (*N* = 70,560)	Mean	2.15	1.98	1.80	1.83	1.73
	*N*	7,704	15,358	24,575	6,328	16,595
	Highest					
	qualification	None	Other	O level	A level	Higher
UNSOC (*N* = 13,817)	Mean	2.28	2.07	1.94	1.79	1.83
	*N*	5,328	1,552	2,322	902	3,713
*Notes:* Respondents aged 40 and over. *Source:* Based on Fertility and Family Surveys (FFS), International Social Survey Programme (ISSP), Generations and Gender Programme (GGP), and Understanding Society Survey (UNSOC).						

The availability of individual-level, nationally representative studies permits a comprehensive assessment of the relationship of sibship size and completed fertility. In order to compare the magnitude of such effects and to investigate how far intergenerational patterns may be attenuated by the inclusion of socioeconomic variables, regression models described in the Methods section were fitted to those individuals aged 40 and over. The main variable used was education level, together with religious participation, as recorded in the ISSP data analyses.

The first set of analyses aims to identify the average contribution of family size and other variables in each of the datasets using an increasingly comprehensive series of models in Table 3, as set out in the Methods section. All models were fitted to datasets that excluded missing education values to enable analysis of deviance; the sample sizes are shown in the “Complete *N*” columns in Appendix A. Inclusion of a sibling coefficient in Model 2 substantially improves the fit, as does inclusion of education (and the religious commitment variable from the ISSP data) in Model 3. However, including an interaction term for siblings by sex in Model 4 does not significantly improve the fit in any of these cases, suggesting that the relationship of parents' fertility with that of their sons and daughters is similar. The coefficients for the preferred Model 3, apart from the country coefficients, are shown in Table 4. The sibling coefficients are generally smaller than that of the other controls, since they represent the marginal effect per additional sibling. After controlling for age, sex, and country, all the other coefficients are statistically significant and in the expected direction, with lower education, higher religious attendance, and larger number of siblings all associated with higher fertility. Since these socioeconomic variables are intergenerationally transmitted, failure to control for them would lead to Pearson, Lee, and Bramley-Moore's (1899) “spurious” relationships.

**Table 3:** Analysis of deviance for quasipoisson models, respondents aged 40 and over, alternative data sources

				Analysis of deviance compared with previous model			
Survey	Model	Residual df	Residual deviance	df	Deviance	F-stat	Pr(>F)
ISSP (*N* = 12,617)	Model 1	12,596	13,245.2	−	−	−	−
	Model 2	12,595	13,079.0	1	166.1	177.0	<.000001
	Model 3	12,587	12,935.0	8	144.0	19.4	<.000001
	Model 4	12,586	12,934.8	1	0.2	0.2	0.63
FFS (*N* = 18,011)	Model 1	17,998	15,402.8	−	−	−	−
	Model 2	17,997	15,192.2	1	210.6	300.7	<. 000001
	Model 3	17,993	15,087.9	4	104.3	37.7	<.000001
	Model 4	17,992	15,085.5	1	2.4	3.5	0.06
GGP (*N* = 70,560)	Model 1	54,493	50,614.5	−	−	−	−
	Model 2	54,492	49,718.4	1	896.1	1153.5	<.000001
	Model 3	54,488	49,243.9	4	474.4	156.0	<.000001
	Model 4	54,487	49,243.9	1	0.0	0.0	0.93
UNSOC (*N* = 13,817)	Model 1	13,814	15,974.9	−	−	−	−
	Model 2	13,813	15,674.2	1	300.7	318.7	<. 000001
	Model 3	13,809	15,570.0	4	104.2	27.9	<.000001
	Model 4	13,808	15,569.7	1	0.3	0.3	0.56
*Notes:* Model 1: Children = Country + Sex + Age; Model 2: Children = Country + Sex + Age + Sibs; Model 3: Children = Country + Sex + Age + Sibs + Educ; Model 4: Children = Country + Sex + Age + Sibs + Educ + Sibs:Sex. *Source:* Based on Fertility and Family Surveys (FFS), International Social Survey Programme (ISSP), Generations and Gender Programme (GGP), and Understanding Society Survey (UNSOC).

**Table 4:** Coefficients of quasipoisson model for number of children, respondents aged 40 and over, alternative data sources

	Survey			
Variable	ISSP	FFS	GGP	UNSOC
Female (ref male)	0.001	0.070***	0.018**	0.095***
Age (in decades)	0.062***	0.102***	−0.010***	0.051***
Sibs	0.032***	0.029***	0.037***	0.045***
Education (ref Group 1)				
Group 2	−0.068***	−0.102***	−0.111***	−0.046*
Group 3	−0.142***	−0.155***	−0.194***	−0.091***
Group 4		−0.125***	−0.199***	−0.170***
Group 5		−0.213***	−0.253***	−0.149***
Religious attendance (ref weekly)				
2–3 times a month	−0.047*			
Once a month	−0.084**			
Several times a year	−0.109***			
Less frequently a year	−0.123***			
Never	−0.163***			
No reply	−0.101**			
*Notes:* *** *p* <.001; **p < .01; *p < .05. Education groups as in Table 2: ISSP: Group 1 (ref) bottom; Group 2 middle; Group 3 top. FFS & GGP: Group 1 (ref) primary; Group 2 lower secondary; Group 3 upper secondary; Group 4 postsecondary nontertiary; Group 5 tertiary. UNSOC: Group 1 (ref) none; Group 2 other; Group 3 O level; Group 4 A level; Group 5 higher. *Source:* Based on Fertility and Family Surveys (FFS), International Social Survey Programme (ISSP), Generations and Gender Programme (GGP), and Understanding Society Survey (UNSOC).				

The coefficients for education level vary between countries, so in order to estimate their maximum contribution to the explanation of differences in fertility, separate models were fitted to each country, as set out in the Methods section. Sibling coefficients were estimated from quasipoisson models for each country, (a) including age and sex only (unadjusted for socioeconomic factors) and (b) also including education level and—in the case of the ISSP—religious attendance (adjusted models); see Figure 3 and Appendix B. All coefficients are positive, and the great majority are statistically significant at the 5 percent level. The rankings of country sibling regression coefficients and the overall correlation coefficients are very similar (see Figures 1 and 3).

**Figure:**
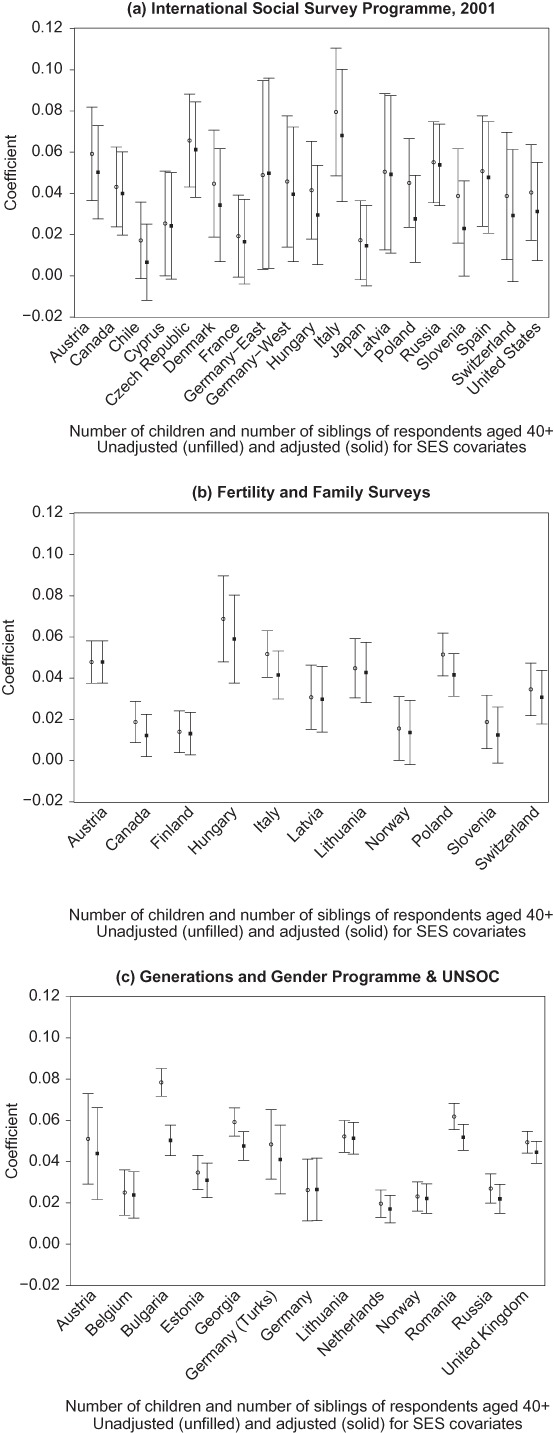
**Figure 3.**  Regression coefficient for number of siblings, unadjusted and adjusted for socioeconomic covariates; respondents aged 40 and over.

Values for the four datasets in Figure 3 are consistent. The estimated values for the coefficients in the unadjusted models show that each additional sibling is associated with around 4 percent higher fertility among respondents aged 40 and over. As would be expected, the sibling coefficient values reduce after adjustment, but the changes are relatively small and usually remain statistically significant. The median unadjusted sibling coefficient value based on one observation per country dataset presented in Appendix B is 0.040, and the corresponding adjusted value is 0.031; the adjusted coefficient is smaller than the unadjusted one in 29 of the 31 cases. Since variables such as education reflect wider socioeconomic differences, we therefore conclude that the intergenerational fertility association is reduced but is likely to be robust to the inclusion of additional covariates.

## Summary and Conclusions

A limitation of an analysis such as this is that it cannot satisfactorily address the question of the pathways for transmission. For example, age at partnership has been found to be an important proximate determinant of the fertility correlation between generations, and the intergenerational relationship of age at first birth tends to be rather stronger than it is for overall fertility. However, early age at partnership is also associated with higher parental partnership breakdown, and, for example, higher proportions of couples in developed countries such as Britain now divorce as have three or more children, so there is scope for partnership as well as fertility history to become an increasingly important factor, even though high proportions of births now take place outside formal marriage in many developed countries.

“Socialization” is widely cited as the primary mechanism for intergenerational fertility transmission in contemporary societies, although the empirical basis for doing so is weak, as alternative explanations are rarely investigated. Since children are usually socialized by their biological parents over a long and formative period, it is difficult to establish the relative contribution of attitudes, preferences, and social learning. There may be a genetic inherited propensity for continuities in family size (Bras, Van Bavel, and Mandemakers 2013; Huestis and Maxwell 1932:77; Rodgers, Kohler, and Christensen 2002), but once more, few studies are able to distinguish between genetic and socialization factors and their interactions.

Contemporary data show that intergenerational transmission of health status has only a small role to play (impaired fecundability is considered a health problem), given the similarity of patterns for men and women. Transmission of poor obstetric practices and short breastfeeding periods in some families would be expected to lead to intergenerational fertility correlations in overall fertility (although not necessarily in effective fertility) in high-mortality societies. However, current low levels of early age mortality mean that such factors have little impact.

Therefore, for contemporary industrialized country populations, biological ability is a less relevant mechanism, at least outside of specialized groups such as women who choose to start childbearing at older ages, when interactions between behavioral factors and reduced fecundability may be important. In contrast to earlier studies that assumed that any relationship was only likely to be found in noncontracepting populations, it is more plausible that intergenerational fertility continuities will be manifested in post-transitional ones, and that any likely genetic mechanism will be behavioral rather than physiological.

Analysis of large numbers of countries with coordinated datasets provides considerable additional insight into levels, trends, and the contribution of alternative covariates. A number of studies have shown that persistent intergenerational fertility correlations have substantial impacts on the distribution of genetic characteristics, especially those that are important for inherited disease (Austerlitz and Heyer 1998) and overall population growth (Norden 1996). Members of large families are overrepresented in subsequent generations, and in turn, each one has more descendants on average than individuals from smaller families. Therefore, intergenerational continuities in fertility behavior play a substantial role in keeping fertility and population growth higher than they would be in the absence of such transmission (Murphy and Wang 2002). The existence of such correlations has wider social implications at the family level. People with higher numbers of siblings are likely to have more children, but also more nephews, nieces, aunts, and uncles than individuals from small families. Therefore, such correlations will tend to increase the variability in the size of kin networks.

Since existence of wider kin networks has been identified as relevant to childbearing in both historical and contemporary developed and developing countries (e.g., Bernardi and White 2010; Sear, Mace, and McGregor 2003; Tymicki 2004), the impact of intergenerational fertility transmission extends beyond parent-child associations. Intergenerational fertility continuities have been both persistent and increasing in developed countries over the twentieth century, but there are arguments that these associations would be expected to be largest when fertility is declining, as the low fertility of “early adopters” would be continued by their own children, whereas more traditional groups would maintain higher fertility, but that such effects would be less substantial in more stable periods. The strength of the relationship does not appear to have increased in the most recent period analyzed here, instead remaining largely constant. However, I do not find evidence of weakening intergen**e**rational fertility transmission over time. This is in contrast to many of the “traditional” fertility differentials, such as religion or urban/rural residence, which have declined or disappeared over time. Fertility analysis is often concerned with identifying variables associated with fertility decisions and outcomes, and intergenerational continuities at the individual family level retain a strong influence.

## Appendix

**Appendix A.** Summary statistics: mean number of siblings, children, age of respondent, and total and nonmissing sample sizes, by sex of respondent; International Social Survey Programme 2001 (ISSP

		Male					Female			
Country	Siblings	Children	Age	Total *N*	Complete *N*	Siblings	Children	Age	Total *N*	Complete *N*
Austria	2.17	2.21	60.66	295	295	2.05	2.12	60.21	441	441
Canada	3.38	2.57	56.50	356	341	3.61	2.53	54.12	335	325
Chile	4.00	3.03	56.55	484	454	4.29	2.91	55.94	319	306
Cyprus	2.03	2.45	53.40	272	264	2.22	2.57	52.90	231	214
Czech Republic	1.49	1.97	55.29	257	253	1.58	1.96	55.95	382	375
Denmark	2.15	2.17	57.41	359	262	2.15	2.32	59.13	426	294
France	2.43	2.25	59.97	432	422	2.49	2.36	55.58	385	369
Germany–East	2.09	2.29	55.33	120	120	1.82	2.13	55.62	126	126
Germany–West	1.98	2.00	57.01	248	245	2.03	1.99	58.34	282	281
Hungary	1.87	1.94	59.57	436	431	1.60	1.92	61.28	636	633
Italy	1.74	1.82	57.45	269	261	1.89	1.83	58.15	287	283
Japan	2.81	2.05	57.88	402	347	2.99	2.24	58.72	474	402
Latvia	1.39	1.76	55.42	231	215	1.36	1.72	55.16	340	322
Northern Ireland	2.88	2.29	60.42	343	0	2.66	2.40	59.41	517	0
Poland	2.33	2.11	56.16	324	322	2.47	2.23	58.17	466	454
Russia	1.94	1.78	55.04	485	464	1.81	1.68	57.62	668	622
Slovenia	2.42	2.09	54.84	264	260	2.32	2.26	59.06	349	342
Spain	2.49	2.05	58.10	308	276	2.59	2.55	60.13	364	308
Switzerland	2.15	1.73	58.68	336	320	2.38	1.79	59.36	322	302
United States	2.97	2.02	56.78	317	314	2.99	2.18	57.20	354	352
Total	2.41	2.15	57.30	6,538	5,866	2.34	2.16	57.97	7,704	6,751
*Source:* Based on 2001 International Social Survey Programme (ISSP).										

## Appendix

**Table:** Summary statistics: mean number of siblings, children, age of respondent, and total and nonmissing sample sizes, by sex of respondent; Fertility and Family Surveys (FFS)

	Male					Female				
Country	Siblings	Children	Age	Total *N*	Complete *N*	Siblings	Children	Age	Total *N*	Complete *N*
Austria	2.56	1.88	46.70	507	507	2.62	2.16	46.64	1,664	1,662
Canada	3.71	1.73	46.21	1,318	1,261	3.74	1.98	46.41	1,401	1,341
Finland	3.30	1.91	46.82	728	648	3.35	1.99	44.92	1,518	1,437
Hungary	1.96	1.77	42.06	370	369	1.97	1.92	40.06	223	223
Italy	2.99	1.67	44.34	345	295	2.88	1.92	44.38	1,445	1,297
Latvia	2.00	1.75	44.28	422	419	2.09	1.77	44.19	857	850
Lithuania	2.45	1.78	44.30	532	525	2.73	1.76	44.29	770	768
Norway	2.52	2.14	42.00	764	652	2.49	2.22	42.00	547	486
Poland	2.97	2.17	45.52	1,460	1,443	2.95	2.24	44.00	1,248	1,241
Slovenia	2.62	1.91	42.08	384	343	2.79	2.05	42.02	525	482
Switzerland	2.74	1.84	44.20	674	553	2.69	1.82	44.31	1,289	1,209
Total	2.88	1.91	44.81	7,504	7,015	2.89	1.99	44.65	11,487	10,996
*Source:* Based on Fertility and Family Surveys (FFS).										

## Appendix

**Table:** Summary statistics: mean number of siblings, children, age of respondent, and total and nonmissing sample sizes, by sex of respondent; Generations and Gender Programme (GGP)

	Male					Female				
Country	Siblings	Children	Age	Total *N*	Complete *N*	Siblings	Children	Age	Total *N*	Complete *N*
Austria	2.51	1.54	42.20	524	524	2.66	1.79	42.25	783	783
Belgium	3.03	1.69	56.67	2,251	2,240	3.02	1.81	56.13	2,380	2,375
Bulgaria	1.83	1.74	56.82	2,994	2,991	1.89	1.80	55.32	3,284	3,282
Estonia	2.24	1.88	57.17	1,724	1,724	2.37	1.94	58.37	3,321	3,321
France	3.24	1.95	57.22	2,796	0	3.23	2.03	57.18	3,518	0
Georgia	2.73	2.20	55.98	2,488	2,488	2.83	2.16	56.83	3,455	3,455
Germany	1.98	1.51	57.25	2,993	2,944	2.06	1.68	56.23	3,363	3,315
Germany (Turks)	4.13	2.23	52.13	802	720	4.35	2.37	51.05	670	610
Italy	2.51	1.51	51.44	2,626	2,337	2.63	1.69	52.61	3,134	2,839
Lithuania	2.37	1.64	57.92	2,984	2,984	2.45	1.63	58.24	3,140	3,140
Netherlands	3.74	1.99	55.48	2,233	2,229	3.73	2.10	55.78	2,886	2,881
Norway	2.67	2.11	57.03	4,360	4,342	2.61	2.16	56.61	4,513	4,508
Romania	2.75	1.82	57.68	3,864	3,864	2.86	1.93	58.96	4,086	4,086
Russia	2.37	1.72	55.86	2,381	2,223	2.57	1.74	57.32	4,565	4,355
Total	2.64	1.82	56.17	35,020	31,610	2.70	1.90	56.38	43,098	38,950
*Source:* Based on Generations and Gender Programme (GGP) data.										

## Appendix

**Table:** Summary statistics: mean number of siblings, children, age of respondent, and total and nonmissing sample sizes, by sex of respondent; Understanding Society Survey (UNSOC)

Male					Female				
Siblings	Children	Age	Total *N*	Complete *N*	Siblings	Children	Age	Total *N*	Complete *N*
2.04	1.94	58.66	6,039	6,034	2.08	2.13	58.42	7,789	7,783
*Source:* Based on Understanding Society Survey (UNSOC).									

## Appendix

**Appendix B.** Sibling coefficients unadjusted and adjusted for socioeconomic variables

	Unadjusted		Adjusted		
Population	Coefficient	SE	Coefficient	sSE	Source
Austria	0.051	0.011	0.044	0.011	GGP
Belgium	0.025	0.006	0.024	0.006	GGP
Bulgaria	0.078	0.003	0.050	0.004	GGP
Canada	0.019	0.005	0.012	0.005	FFS
Chile	0.017	0.009	0.007	0.009	ISSP
Cyprus	0.025	0.013	0.024	0.013	ISSP
Czech Republic	0.066	0.012	0.061	0.012	ISSP
Denmark	0.045	0.013	0.034	0.014	ISSP
Estonia	0.035	0.004	0.031	0.004	GGP
Finland	0.014	0.005	0.013	0.005	FFS
France	0.019	0.010	0.017	0.010	ISSP
Georgia	0.059	0.004	0.048	0.004	GGP
Germany	0.026	0.008	0.027	0.008	GGP
Germany (Turks)	0.048	0.009	0.041	0.009	GGP
Germany–East	0.049	0.023	0.050	0.024	ISSP
Germany–West	0.046	0.016	0.040	0.017	ISSP
Hungary	0.069	0.011	0.059	0.011	FFS
Italy	0.052	0.006	0.042	0.006	FFS
Japan	0.017	0.010	0.015	0.010	ISSP
Latvia	0.031	0.008	0.030	0.008	FFS
Lithuania	0.052	0.004	0.051	0.004	GGP
Netherlands	0.020	0.003	0.017	0.003	GGP
Norway	0.023	0.004	0.022	0.004	GGP
Poland	0.051	0.005	0.042	0.005	FFS
Romania	0.062	0.003	0.052	0.003	GGP
Russia	0.027	0.004	0.022	0.004	GGP
Slovenia	0.019	0.007	0.012	0.007	FFS
Spain	0.051	0.014	0.048	0.014	ISSP
Switzerland	0.035	0.007	0.031	0.007	FFS
United Kingdom	0.049	0.003	0.045	0.003	UNSOC
United States	0.040	0.012	0.031	0.012	ISSP
*Notes:* Only one value per population presented, in order of UNSOC, GGP, FFS and ISSP.					
*Source:* Based on author's analysis of Fertility and Family Surveys (FFS), International Social Survey Programme (ISSP), Generations and Gender Programme (GGP), and Understanding Society Survey (UNSOC).					
